# Mechanism of traditional Chinese medicine in treating erectile dysfunction: A review

**DOI:** 10.1097/MD.0000000000047652

**Published:** 2026-02-13

**Authors:** Ruoran Zhang, Jingkai Wang, Peng Xu, Hailuo Wang, Kun Pang

**Affiliations:** a School of Medicine, Jiangsu University, Zhenjiang, Jiangsu, China; b The Second Affiliated Hospital of Nanjing Medical University, Nanjing, Jiangsu, China; c Department of Urology, Xuzhou Central Hospital, Nanjing University of Chinese Medicine, Southeast University, Xuzhou Clinical School of Xuzhou Medical University, Xuzhou, Jiangsu, China; d Department of Urology, Peixian People’s Hospital, Jiangsu, China.

**Keywords:** erectile dysfunction, mechanism, therapy, traditional Chinese medicine

## Abstract

Erectile dysfunction (ED) is a common and serious condition. As people’s lifestyles change and the aging of the population, the incidence of ED is increasing. traditional Chinese medicine (TCM) has long been a traditional clinical approach for treating ED in China and has been shown to be effective and safe. A large amount of studies on TCM for ED have revealed various therapeutic mechanisms; however, few reviews have summarized the TCM therapeutic mechanisms currently available for the treatment of ED. Therefore, a systematic analysis of the therapeutic mechanisms of TCM is of great academic and clinical value. Summarizing the molecular mechanisms of TCM for ED from multiple directions enriches the theory of TCM for ED and establishes the clinical significance and development prospect of TCM for ED. It provides basic theoretical support for the discovery and utilization of TCM resources, and provides up-to-date and comprehensive insights into the clinical application of TCM for the treatment of ED. This knowledge may pave the way for the development of more effective drugs and methods.

## 1. Introduction

Erectile dysfunction (ED) is a condition in which the penis fails to achieve or maintain full erection under normal sexual stimulation, making it unable for the patient to have normal sexual intercourse.^[1]^ ED is now a common disease, with an increasing incidence due to the accelerated pace of life, increased stress, and changes in lifestyle.^[2]^ According to statistics, about 150 million men worldwide suffer from this disease, accounting for 20% of the world’s adult males, and the statistics are increasing year by year.^[3]^ The total prevalence of ED in Chinese men is about 26.1%, of which the prevalence in men over 40 years old is as high as 40.2%.^[4]^

Following sexual stimulation, nitric oxide (NO) is released from nonadrenergic cholinergic nerve fibers and acetylcholine from parasympathetic cholinergic nerve fibers. The release of these 2 hormones increases concentrations of cyclic guanosine monophosphate (cGMP) in the vasculature and decreases intracellular Ca^2+^ levels, leading to smooth muscle cell relaxation. When the smooth muscle relaxes, blood can fill the cavernous space of the corpus cavernosum to pressurize the small veins, blocking venous blood outflow to cause penile erection. When phosphodiesterase type 5 (PDE-5) is hydrolyzed, the process is reversed and ED occurs.^[5]^ This is the mechanism by which PDE-5 inhibitors are used to treat ED. PDE-5 inhibitors are also the 1st line of treatment for ED, and have become the mainstay of treatment because of their short-term efficacy, but their long-term efficacy is poor.^[6]^ In addition, PDE-5 inhibitors are ineffective in 35% of ED patients and are prone to headaches, myalgia, and other painful side effects.^[7]^ Recent reports suggest that PDE-5 inhibitors may cause hearing loss with severe hypotension.^[8,9]^

As a result, the use of drugs is severely limited, especially in patients with cardiovascular disease. Other treatment options mainly include intracorporeal injections of vasoactive drugs, vacuum suction devices, and penile prosthesis implants. These invasive treatments may cause pain and many adverse effects in patients.^[10]^ Although there are many treatments available for ED, the efficacy of these therapies is uncertain, symptoms tend to recur, and many patients find it unbearable. Moreover, as life expectancy increases in the general population, the healthcare burden and quality of life issues associated with ED are expected to be quite significant.^[11]^

In this situation, various alternative or complementary treatments are being investigated to treat ED more effectively. In China, traditional Chinese medicine (TCM) has been widely used to treat ED for more than 2000 years, and doctors have accumulated a wealth of experience. TCM is highly recognized for its good efficacy and few adverse reactions. Moreover, TCM has unique advantages in treating ED, which can regulate the overall state of patients while improving their symptoms in order to achieve both symptomatic and curative effects.^[12]^ Both acupuncture and herbs are believed to have significant effects on ED, and it significantly improves erectile function for ED patients.^[13]^

Acupuncture, as one of the representative treatments, has received more and more attention for the advantages of stable efficacy, low recurrence rate, and small adverse effects. The selection of acupoints and the pattern of combinations are the key to the clinical efficacy of acupuncture in the treatment of ED.^[14]^ Chinese herbs have been widely used in the treatment of ED in China for thousands of years. It has been reported that the combination of Chinese herbs with PDE-5 inhibitors is more effective and has fewer adverse effects than PDE-5 inhibitors alone.^[15,16]^ Recent studies have found that the effective active ingredients in TCM can not only increase the activity of cGMP, but also improve the blood supply of peripheral blood vessels, thus achieving the purpose of treating ED.

There are many factors that affect the pathophysiology of ED, including vascular, neurologic, and hormonal abnormalities, which are all major contributors to the development of ED.^[5]^ TCM is characterized by multi-component, multi-target, and multi-pathway synergistic effects in the treatment of ED. This is also consistent with the systemic or whole view of TCM theory.^[12]^ On this basis, researchers still need to find effective multi-target combination therapy. Therefore, in this review, we will start from a molecular perspective and focus on the mechanism of action of multi-pathway therapy on ED pathophysiology. The aim is to comprehensively review the current molecular evidence for ED treatment in TCM and provide more adequate options for clinical treatment of ED, with a view to benefiting clinical treatment and basic research.

## 2. Acupuncture treatment for ED

In China, acupuncture is an irreplaceable traditional treatment method that has been passed down for more than 2000 years and has been recognized and accepted by the Chinese people. WHO recommends acupuncture for a wide range of applications, including diseases of the musculoskeletal system, neurological disorders, gynecological disorders, skin injuries, and psychological disorders.^[17,18]^ Contemporary research suggests that the neurophysiological effects of acupuncture are the result of central nervous system activation and neurotransmitter modulation. Based on these mechanisms, acupuncture may be an important treatment for ED. Chinese medicine has a long history of recognizing and treating ED, and has accumulated a wealth of experience that is highly recognized for its efficacy. For some patients, acupuncture may even be cheaper and more effective than traditional therapies, thus making it a promising option for the treatment of ED.

Current experiments have found that the mechanisms related to the improvement of ED by acupuncture are: some functional areas of the hypothalamus in ED patients may have abnormalities, and acupuncture can improve male ED through hypothalamic regulation.^[19]^ Effective treatment of psychogenic ED by modulating the HPA axis to relieve patients’ anxiety and depression.^[20]^ Acupuncture can regulate testosterone levels in men and improve clinical symptoms in ED patients.^[21]^ Acupuncture can significantly increase NO levels in penile tissues and promote vasodilatation and congestion of the corpus cavernosum, thereby improving its erectile function.^[22]^ There is also evidence that although acupuncture can affect the pelvic nerves, it is not clear whether acupuncture is sufficient to locally affect NO release in the corresponding vessels. Whether acupuncture for ED actives the NO–cGMP pathway remains to be further investigated.^[13,23]^

A large number of studies have shown that TCM, including acupuncture, has a high success rate in treating ED. However, a subset of scholars suggests that the currently available evidence is insufficient to demonstrate that acupuncture is an effective intervention for the treatment of ED.^[14]^ The validity of the studies remains questionable mainly due to their small number and variable quality. The exact mechanism of action of acupuncture on ED is still unclear, and future experiments need to better characterize its molecular mechanisms to make an effective case for the effectiveness of acupuncture for ED treatment.^[19,24]^

## 3. Chinese herbal treatment for ED

A large number of traditional Chinese herbal medicines have been used in the treatment of ED in the form of individual applications or compounded formulas, but most herbal therapies are used empirically and are therefore unconvincing. Moreover, the mechanism of action, pharmacokinetics, and therapeutic targets of most herbal medicines remain elusive due to the fact that herbal medicines contain a wide range of chemical constituents and have many nutritional and pharmacological effects. Although Hongjing I granule demonstrated significant improvement in symptoms of patients with mild to moderate ED in a double-blind randomized controlled clinical trial conducted by Run-Nan Xu et al,^[25]^ there remains a paucity of large-scale randomized controlled trials to evaluate the efficacy of TCM in treating ED.^[12,26]^

However, many animal studies and in vitro studies have resulted in various pharmacologic mechanisms of herbal medicines for ED. The biological effects of Chinese herbal medicines for ED are mainly through certain pathways to achieve anti-inflammatory, antioxidant, anti-aging, antifibrotic, regulation of hormones in the body, promotion of NO production, protection of vascular endothelium and nerve cells, and promotion of nerve and vascular endothelial cell regeneration. A small number of herbs improve ED through 1 pathway, while most involve multiple pathways to treat ED.

As mentioned above, these biological pathways are interrelated and these natural products may act in a multi-component and multi-target manner. In this context, modern biotechnology has been utilized to investigate the potential therapeutic mechanisms of these herbs in order to clarify the utility of Chinese herbs for the treatment of ED. We summarized the mechanisms of specific TCM treatments for ED patients or animal models by analyzing and reviewing the results of ex vivo and in vivo experiments. This will help to combine TCM theories with the latest medical science to explore the mechanisms and efficacy in depth, which is of great significance for the treatment of ED with TCM.

## 4. Specific mechanism of Chinese herbal for ED

### 4.1. Promote NO production

The discovery of NO as an intercellular messenger or neurotransmitter opens a new era for identifying important mechanisms of autonomic innervation of organs and tissues. It provides an avenue for the development of new therapies based on new concepts of molecular and cellular interactions.^[27]^ NO, an unstable gaseous molecule, has been shown to be an endothelial relaxing factor that regulates vascular tone, platelet aggregation and adhesion, and vascular smooth muscle proliferation. Later, NO was identified as the nonadrenergic cholinergic neurotransmitter that innervates various smooth muscles, including the corpus cavernosum. NO from nerves and endothelial cells plays a crucial role in initiating and maintaining increased intracavitary pressure, vasodilation, and erection. These are dependent on NO activation of cGMP in smooth muscle cells.^[28,29]^ It was found that NO is catalyzed by nitric oxide synthase (NOS), subtypes neuronal NOS (nNOS), endothelial NOS (eNOS), and inducible NOS and that the endothelial cells of the corpus cavernosum respond to physical and chemical stimuli by increasing each subtype of NOS to promote the production and release of NO.^[30]^ ED is caused by a variety of pathogenic factors, especially impaired formation and action of NO. And to date, supplementation with NO or cGMP promises to be the most promising treatment for ED patients.^[31,32]^ By summarizing the latest research progress of Chinese medicines in promoting NO production in various ED animal models, mainly including the subtypes of NOS and related pathways, and the related proteins that promote NO production. We have demonstrated the science of Chinese medicines in treating ED and provided a detailed understanding of the therapeutic mechanism of Chinese medicines. It demonstrates the scientific validity of the treatment of ED with Chinese medicines and allows more people to understand the therapeutic mechanism of Chinese medicines in detail. It is hoped that more cost-effective Chinese medicines than PDE-5 inhibitors such as sildenafil can be found to provide more therapeutic options for clinicians and ED patients.

Yi-No Wu et al^[33]^ reported that erectile function could be improved in cavernous nerve injury (CNI)-ED rats with *Ginkgo biloba* extract (GBE). High-dose GBE treatment increased nNOS levels and promoted NO production. In addition, it was found that high-dose GBE increased the survival rate of neurons after CNI and enhanced the neuroprotective effect of erectile function in CNI-ED rats. Shuai Liu et al^[34]^ found in their study that fermented *Gynochthodes officinalis* active PI3K/Akt/eNOS pathway to ameliorate erectile response of diabetes mellitus ED (DMED) rats. James Chen et al^[35]^ found that Osthole promotes rabbit corpus cavernosum smooth muscle relaxation by inhibiting phosphodiesterase, releasing NO, and enhancing cGMP and cAMP signaling. F Lin et al^[36]^ found in their experiments that Panax notoginseng saponins could elevate the content of eNOS and cGMP in the cavernous tissue of DMED rats, and improve the erectile function of DMED rats through the NO/cGMP pathway. Several studies^[37,38]^ have shown that the main mechanism of action of icariin in the treatment of ED rats is to promote the expression of 3 NOS in the cavernous tissue, enhance the activity of the NO/cGMP signaling pathway, and promote NO production. Hui Fu et al^[39]^ found that *Eucommia ulmoides* Oliv. leaf extract (EULE) could activate the Akt–eNOS pathway, increase NO production, and restore erectile function in DMED rats. Yueyang Zhang et al^[40]^ found that quercetin protected the expression and function of eNOS in the cavernous endothelial cells of arterial ED rats and restored part of the normal function of the NO–cGMP pathway during penile erection. Shiting Yu et al^[41]^ found in their study that Chinese yam cold-soaking extract elevated inducible NOS content and stimulated NOS/cGMP pathway activity in rat cavernous cells.

Many other formulas have been used to treat ED in China, and several studies have demonstrated that most of them have ability to activate NO/cGMP, which facilitates ED recovery by promoting the release of NOS from the endothelial cells of the corpus cavernosum. These formulas include *Achyranthes bidentate* radix plus semen vaccariae, Wubi Shanyao Pills, leech and centipede granules (LCG), Yidiyin, Shuganyiyang capsule, and Xuefu Zhuyu decoction.^[42–47]^ Furthermore, owing to its diverse chemical constituents, such as those found in Danggui Sini Decoction, this formulation exerts therapeutic effects on ED via multiple molecular targets, including AKT1, ALB, IL6, TNF, TP53, and BCL-2.^[48]^

It is worth pondering whether it could be further investigated in modern medicine as a more effective clinical drug with fewer side effects instead of sildenafil.

### 4.2. Protecting corpus cavernosum cells and anti-cavernous fibrosis

Fibrosis of the penile corpus cavernosum is considered to be an important factor in the development of ED, and it is considered to be the main cause of structural disorders of the corpus cavernosum. Penile fibrosis is caused by multiple factors, including endothelial dysfunction (apoptosis, endothelial cell damage, or aging), oxidative stress, vascular and neurogenic causes.^[49,50]^ CNI and diabetes mellitus cause severe impairment of vascular and nerve function, which can lead to cavernous hypoxia and up-regulation of pro-fibrotic factors, promoting cavernous fibrosis.^[51]^ Increased oxidative stress in the corpus cavernosum and decreased free radical scavenging leads to accumulation of reactive oxygen species. Activation of TGF-β1 by reactive oxygen species leads to progression of cavernous fibrosis.^[52]^ Clinical studies have shown that the poor therapeutic efficacy of PDE-5 inhibitors are related to structural changes in the penis, with endothelial fibrosis limiting the availability of NO especially in patients with diabetes mellitus and CNI.^[53]^ In the context of modern medicine, it is crucial to find alternative drugs with a different mechanism of action than PDE-5 inhibitors to treat ED even in the setting of cavernous fibrosis. In conclusion, the treatment of cavernous fibrosis may be related to inhibitors or drugs that target inflammation, oxidative stress and apoptosis. Several studies have shown that the therapeutic techniques of Chinese medicine hold promise as a new tool in the treatment of ED in cavernous fibrosis.^[49,54]^

Yi-No Wu et al^[33]^ found that GBE could promote cavernous smooth muscle and nerve regeneration, prevent cavernous smooth muscle atrophy effectively, and prevent cavernous fibrosis due to nerve injury in CNI-ED rats. In addition, several studies have shown that GBE can effectively promote nerve repair and regeneration and resist nerve injury.^[55,56]^ Several studies^[36,57]^ have comprehensively demonstrated that Panax notoginseng saponins protects endothelial cell function by antagonizing oxidative stress, eliminating oxidative inhibition of Akt, controlling the accumulation of AGEs, and inhibiting spongiocyte apoptosis. Studies on the therapeutic mechanism of Icariin have found^[40]^ that it can significantly reduce the expression of TGFβ1 and increase the content of rat cavernous smooth muscle. Icariin may reduce apoptosis of rat cavernous smooth muscle and endothelial cells by down-regulation of the TGFβ1–Smad2 signaling pathway, and maintain the integrity of neurons. Liu T et al^[58]^ found that bcl-2, bcl-xl, PECAM-1, and SMA levels and nerve fiber counts were normalized in DMED rats treated with Ginsenoside Rg3. Ginsenoside Rg3 protects the erectile ability of DMED rats by decreasing the apoptotic index of corpus cavernosum cells. Taotao Sun et al^[59]^ found that Paeonol could regulate endothelial dysfunction by inhibiting the HMGB1/RAGE/NF-κB pathway, inflammation, endothelial dysfunction, apoptosis, and fibrotic activity in their experimental study of the mechanism of Paeonol treatment in DMED rats. In their study, Miao-Yong Ye et al^[60]^ found that the mechanism of Salidroside in the treatment of CNI-ED rats mainly includes the promotion of cytoprotective autophagy to inhibit apoptosis and fibrosis, and the prevention of apoptosis in neuronal, endothelial, and corpus cavernosum smooth muscle cells. Another study proposed that Salidroside has a protective effect on hypoxia-induced damage to cavernous smooth muscle cells.^[61]^ The results of several studies^[62,63]^ demonstrated that LCG ameliorated fibrosis by significantly decreasing VCAM-1, ICAM-1, and CD62P. In addition, LCG also modified erectile function in DMED rats by inhibiting oxidative stress and spongy endothelial cell apoptosis and fibrosis through inhibition of the HIF-1α/mTOR pathway, CaSR/PLC/PKC pathway, and TGF-β/Smad pathway. Miao-Yong Ye et al^[52]^ found that Buyang Huanwu Decoction may inhibit apoptosis and fibers of cavernous smooth muscle and restore smooth muscle content by inhibiting the ROS/JNK/c-Jun pathway. Yuanyuan Liu et al^[64]^ demonstrated that Simiao Biejia granules improved oxidative stress damage, vascular endothelial repair, and angiogenesis in the penile tissue of DMED rats. Luteolin, a key component of Simiao Biejia, plays a significant role by activating the PI3K/Akt pathway, regulating the expression of nNOS and NF-κB. Additionally, Pingyu Ge et al^[65,66]^ reported that Quyuhuatanerxian decoction, a TCM formula, could inhibit NLRP3 inflammasome activation, reduce inflammatory and oxidative stress responses, and suppress the activity of the JAK-STAT signaling pathway, ultimately alleviating hyperuricemia-induced ED in rats. Furthermore, Xiao Li et al^[67]^ found that Xuefu Zhuyu decoction promotes vascular endothelial dilation by regulating the related molecules of the CaSR/PLC/PKC and MEK/ERK/RSK pathways, improves the ischemia and hypoxia state of corpus cavernosum smooth muscle, and thereby enhances erectile function in DMED rats. In another study, Jie Wang et al^[68]^ highlighted the therapeutic effects of Radix Paeoniae Rubra and Radix *Angelicae sinensis* Granules in DMED rats by reducing penile hypoxia stress, improving the arrangement of endothelial cells and smooth muscle cells, reversing histopathological damage, and restoring the ratio of smooth muscle to collagen. The study also identified key regulatory pathways, including hypoxia-induced signaling pathways such as HIF-1, MAPK, cAMP, and Ras.

### 4.3. Promote sex hormone secretion

Testosterone is the driving hormone for male sexual development and function. It has roles in the central nervous system, peripheral nervous system, and end organs. Testosterone production declines with age and the prevalence of hypogonadism increases with age.^[69]^ The prevalence of hypogonadism is higher in specific populations, including men with type 2 diabetes, metabolic syndrome, obesity, cardiovascular disease, chronic obstructive pulmonary disease, kidney disease, and cancer. Studies have shown that once testosterone drops below about 230 ng/dL, men begin to experience ED. This is primarily because hypogonadal men have greater endothelial dysfunction, which results from a lack of sex hormones.^[70]^ Clinical trial findings suggest testosterone improves erectile function.^[71]^ Testosterone combined with PDE-5 inhibitors enhances response to PDE-5 inhibitors in patients with ED and hypogonadism.^[72,73]^ In older men with low testosterone levels and low libido, testosterone consistently improves most types of sexual activity, libido, and erectile function. However, the long-term effects of testosterone therapy are unknown and further research is needed.^[69]^ Specific recommendations for clinical practice cannot be made because the overall quality of the current studies is not good enough to draw definitive conclusions about the efficacy and safety of testosterone.^[74]^ However, herbal medicine, as an alternative and complementary treatment, can yield better responses in terms of improved International Index of Erectile Function-5 scores, clinical recovery rates, and testosterone levels, without an increase in side effects.^[26]^

Several studies have^[75–77]^ found a significant increase in testosterone production after treatment with *Tribulus terrestris* in animal and clinical trials. Furthermore, clinical studies have shown a statistically significant correlation between testosterone and International Index of Erectile Function-5. Hui Fu et al^[39]^ found that serum follicle-stimulating hormone, luteinizing hormone, and testosterone concentrations were significantly elevated in DMED rats treated with EULE. The results suggest that EULE has a stimulatory effect on the hypothalamic–pituitary–gonadal axis, but whether it is involved in the improvement of erectile function in DMED rats is still under investigation. Shiting Yu et al^[41]^ found that testosterone secretion and interstitial cell number were significantly reduced in model rats in their experiments. After treatment with Chinese yam cold-soaking extract, testosterone secretion and interstitial cell number increased with the increase of drug concentration, as a way to restore erectile function in kidney-yang deficiency syndrome rats. Palanichamy C et al^[78]^ demonstrated that treatment with *Mimosa pudica* L. extract significantly increased hormone levels (testosterone, follicle-stimulating hormone, and luteinizing hormone) in both serum and testicular homogenates of diabetic rats. Additionally, the extract enhanced aphrodisiac performance in rats by increasing antioxidant enzyme activity. Similarly, studies have shown that aqueous extracts of *Corynanthe pachyceras*^[79]^ and *Schumanniophyton magnificum*^[80]^ can significantly elevate serum testosterone levels, thereby improving erectile function in rats. In a clinical trial, a combination of *Punica granatum* fruit rind and *Theobroma cacao* seed extracts was found to enhance sexual function in elderly men by increasing serum testosterone levels.^[81]^

### 4.4. Ion channel blocker

The cavernous smooth muscle is located in the walls of the vascularized sinusoids of the penis and plays a key role in penile erection. Most of the time, the cavernous smooth muscle contracts to minimize blood flow into the sinus cavity; however, in response to sexual stimulation, the cavernous smooth muscle relaxes, allowing the sinus cavity to dilate and fill with blood. Failure of cavernous relaxation leads to ED.^[82]^ The cavernous smooth muscle exhibits spontaneous and coordinated contraction and relaxation, a process that is regulated by the control of Ca^2+^ channels and other ions in the human corpus cavernosum. Therefore, ion channels on the corpus cavernosum are also considered as potential targets for the treatment of ED.^[83]^

Ji-Hong Liu et al^[84]^ showed that tetrandrine inhibits Ca^2+^ influx from extracellular sites by voltage-activated Ca^2+^ tracts. At high concentrations, tetrandrine inhibited the release of cytoplasmic calcium pools in cavernous smooth muscle cells. In this way, it promotes the relaxation of cavernous smooth muscle and improves erectile function in rabbits. Jun Chen et al^[85]^ in their study found that neferine inhibited extracellular Ca^2+^ inward flow and the release of intracellular stored Ca^2+^ from the corpus cavernosum to achieve relaxation of rabbit corpus cavernosum smooth muscle.

According to the above article, we summarized the reports on the animal studies in ED with TCM in Table 1.

**Table 1:** A summary of relevant animal studies is presented.

Intervention	Animals	Mechanism	References
GBE	CNI-ED rats	Increase nNOS and reduce the apoptotic index	Yi-No Wu et al^[33]^
FGO	DMED rats	Active PI3K/Akt/eNOS pathway	Shuai Liu et al^[34]^
Osthole	Rabbits	Increase NO and enhance cGMP and cAMP signal	James Chen et al^[35]^
PNS	DMED rats	Activate NO/cGMP pathway	F Lin et al^[36]^
Icariin	DMED rats	Increase NO and inhibite TGFβ1/Smad2 pathway	Tao Liu et al^[37]^
EULE	DMED rats	Activate Akt-eNOS pathway and benefit the HPG axis	Hui Fu et al^[39]^
Quercetin	A-ED rats	Activate NO/cGMP pathway	Yueyang Zhang et al^[40]^
CYCSE	KDS-Yang rats	Activate the NO/cGMP pathway, enhance Nrf2/HO-1 pathway and inhibite TGF- β1/SMAD2/3 pathway	Shiting Yu et al^[41]^
LCG	DMED rats	Increase NO and inhibit CaSR/PLC/PKC pathway	Jian Xiong Ma et al^[46]^
BHD	CNI-ED rats	Inhibit fibrosis of CC and ROS/JNK/c-Jun pathway	Miao-Yong Ye et al^[52]^
PNS	DMED rats	Increase Akt expression, suppressed oxidative stress	Huan Li et al^[57]^
Ginsenoside Rg3	DMED rats	Reduce apoptotic index in corpus cavernosum cells	Tao Liu et al^[58]^
Pae	DMED rats	Inhibit HMGB1/RAGE/NF-κB pathway, inflammatory, apoptosis	Taotao Sun et al^[59]^
Sal	CNI-ED rats	Inhibit apoptosis and fibrosis	Miao-Yong Ye et al^[60]^
Sal	Rats	Reduce Expression of Cx43 in Corpus Cavernosum smooth muscle cells	Jianfeng Zhao et al^[61]^
LCG	DMED rats	Activate the NO/cGMP/PKG pathway, PI3K/Akt/nNOS pathway, cAMP/PKA pathway, and inhibit the HIF-1α/mTOR pathway	Jian Xiong Ma et al^[63]^
SMBJ	DMED rats	Activate PI3K/Akt/nNOS pathway and inhibit NF-κB pathway	Yuanyuan Liu et al^[64]^
QYHT	HUA-ED rats	Inhibit inflammation, oxidative stress, JAK-STAT pathway	Pingyu Ge et al^[66]^
XFZYD	DMED rats	Activate CaSR/PLC/PKC and MEK/ERK/RSK pathway	Xiao Li et al^[67]^
RAG	DMED rats	Activate eNOS/iNOS/HMOX1 pathway and inhibit HIF-1α pathway	Jie Wang et al^[68]^
Mimosa pudica L. extract	DMED rats	Inhibit oxidative stress and PDE-5 enzyme	Palanichamy C et al^[78]^
CPE	Rats	Increase NO and Inhibit oxidative stress	Ndomgang AJ et al^[79]^
Tet	Rabbits	Inhibit the Ca^2+^ influx from the extracellular	Ji-Hong Liu et al^[84]^
Nef	Rabbits	Inhibit of extracellular Ca^2+^ influx and the release of intracellular stored Ca^2+^	Jun Chen et al^[85]^

A-ED = arterial erectile dysfunction, BHD = Buyang Huanwu Decoction, CNI = cavernous nerve injury, CPE = aqueous extracts of *Corynanthe pachyceras*, CYCSE = Chinese yam cold-soaking extract, DMED = diabetes mellitus erectile dysfunction, ED = erectile dysfunction, EULE = *Eucommia ulmoides* Oliv. leaf extract, FGO = fermented *Gynochthodes officinalis*, GBE = *Ginkgo biloba* extract, HPG = hypothalamic–pituitary–gonadal, HUA = hyperuricemia, iNOS = inducible NOS, KDS-Yang = kidney-yang deficiency syndrome, LCG = leech and centipede granules, Nef = neferine, Pae = Paeonol, PNS = Panax notoginseng saponins, QYHT = Quyuhuatanerxian decoction, RAG = Radix *Angelicae sinensis* granules, Sal = salidroside, SMBJ = Simiao Biejia, Tet = tetrandrine, XFZYD = Xuefu Zhuyu decoction.

## 5. Discussion

The microstructural damage changes in ED mainly focus on apoptosis, fibrosis, oxidative stress, and hypogonadism in smooth muscle and vascular endothelial cells.^[5]^ A large number of preclinical studies have shown that a variety of TCM can treat ED through multiple pathways such as anti-inflammatory, antioxidant, and protection of vascular smooth muscle cells and nerves.^[12]^ TCM treatment of ED has the unique advantages of multi-component, multi-target, and multi-pathway coordinated regulation.

In summary, TCM has potential safety and efficacy in the treatment of ED and remains a promising area of research. However, there are still many challenges to realize the promotion of TCM in the treatment of ED. Most current studies have focused on exploring the efficacy of single active ingredients and their mechanisms of action. Most of the studies related to the treatment of ED by TCM involve only specific types of pathology and signaling pathways, and researchers also need to focus on the cross-cutting and complex nature of TCM. Focusing on only 1 active ingredient of TCM and ignoring its systemic and holistic nature cannot well reflect the specific pharmacological effects of different active ingredients. In future studies, exploring the synergistic effects of multiple natural compounds on the same or different biological targets may be a new research area.^[45,86]^ As a typical representative of combination therapy, TCM formulas have been used to treat various diseases since ancient times. Most TCM formulas consist of a main component and multiple auxiliary components, which assist and facilitate the delivery of the main component. Reasonable drug ratios can maximize the effectiveness of the various components. Currently, there is a lack of research on the ratio of components in TCM formulas, and future research should pay more attention to the ratio of components and combine pharmacological experiments with clinical verification. The endpoints of drug dosage, treatment duration, and therapeutic efficacy assessment of TCM treatment have not been determined. Most of the current studies have been conducted on animals, and there is a lack of extensive phase II and phase III clinical trials, and the widespread use of TCM therapy faces a large number of ethical, legal, and social issues.^[26,87]^ It is noteworthy that most of the active ingredients of TCM have not yet been developed for clinical use, although pharmacological studies have shown that they are safe and effective. Some studies have reported that certain types of herbal medicines can cause liver or kidney damage. Therefore, the toxicological studies on the active ingredients of herbal medicines for the treatment of ED need to be further improved and supplemented.^[88]^

We believe that the specific mechanism of action of TCM in treating ED can be further clarified and the drawbacks of high cost and inefficiency can be reduced through a large number of preclinical studies, phase II, and phase III clinical trials validation in the near future. We should make every effort to fully prepare for the translation of TCM treatment of ED from preclinical studies to clinical application, and inspire and guide researchers to further explore the mechanism, clinical efficacy and safety of TCM treatment of ED. It will make a great leap in our understanding of TCM science.

## Author contributions

**Conceptualization:** Kun Pang.

**Resources:** Peng Xu, Hailuo Wang.

**Supervision:** Kun Pang.

**Writing – original draft:** Ruoran Zhang, Jingkai Wang, Kun Pang.

**Writing – review & editing:** Kun Pang.
